# Chyloabdomen in critically ill patient after Hartmann’s procedure

**DOI:** 10.1093/jscr/rjac150

**Published:** 2022-05-17

**Authors:** A Torres-Rey, H Soler-Bernardini, G Bolaños-Avila

**Affiliations:** Ponce School of Medicine and Health Sciences, St. Luke's Episcopal Hospital Surgery Department, Ponce, Puerto Rico; Hospital Episcopal San Lucas, General Surgery, Ponce, Puerto Rico; Hospital Episcopal San Lucas, General Surgery, Ponce, Puerto Rico

## Abstract

Post-surgical chylous ascites (CA) is extremely rare in colon surgery, known as the extravasation of creamy fluid rich in triglycerides accumulating in the peritoneal cavity. The incidence of CA after colorectal surgery remains between 1 and 6.5%. A 71-year-old female presented to the Emergency Department complaining of generalized abdominal pain, weakness and anorexia for several days ago and episodes of hematochezia which started the day before admission. Biopsy from colonoscopy revealed mucinous adenocarcinoma. Rectal mass resection with Hartmann’s procedure was performed due to obstructive recto-colonic mass. Pathology report confirmed pT4aN0M0 tumor invading through the visceral peritoneum. On post-operative Day number 4, drainage output increased, changing appearance to a whitish color. The diagnosis of CA was confirmed by obtaining >550 mg per dL of triglycerides. Changes to a high-protein, low-fat with medium chain fatty acids were made to her enteral diet. After 48 hours of diet adjustment, the drainage output of CA resolved.

## INTRODUCTION

Chylous ascites (CA) is known as the extravasation of creamy fluid with a high composition of triglycerides, which accumulates in the peritoneal cavity [1]. It is lymph fluid from the thoracic or abdominal cavity. With a reporting incidence of around 1 per 20 000 case admissions in large university-based hospitals over a 20-year term, it is not a common finding [2]. Other investigators believe that CA incidence has increased due to prolonged survival of cancer patients and more complex retroperitoneal surgeries [2]. There is not vast evidence of CA following colorectal surgery because of its extremely rare occurrence. Despite this, documentation of CA has been more commonly reported following trauma, lymphatic system obstruction and laceration, during pancreatic resections, abdominal aortic aneurysm repair, gynecology pelvic surgery and others [3, 4].

## CASE REPORT

**Figure 1:**
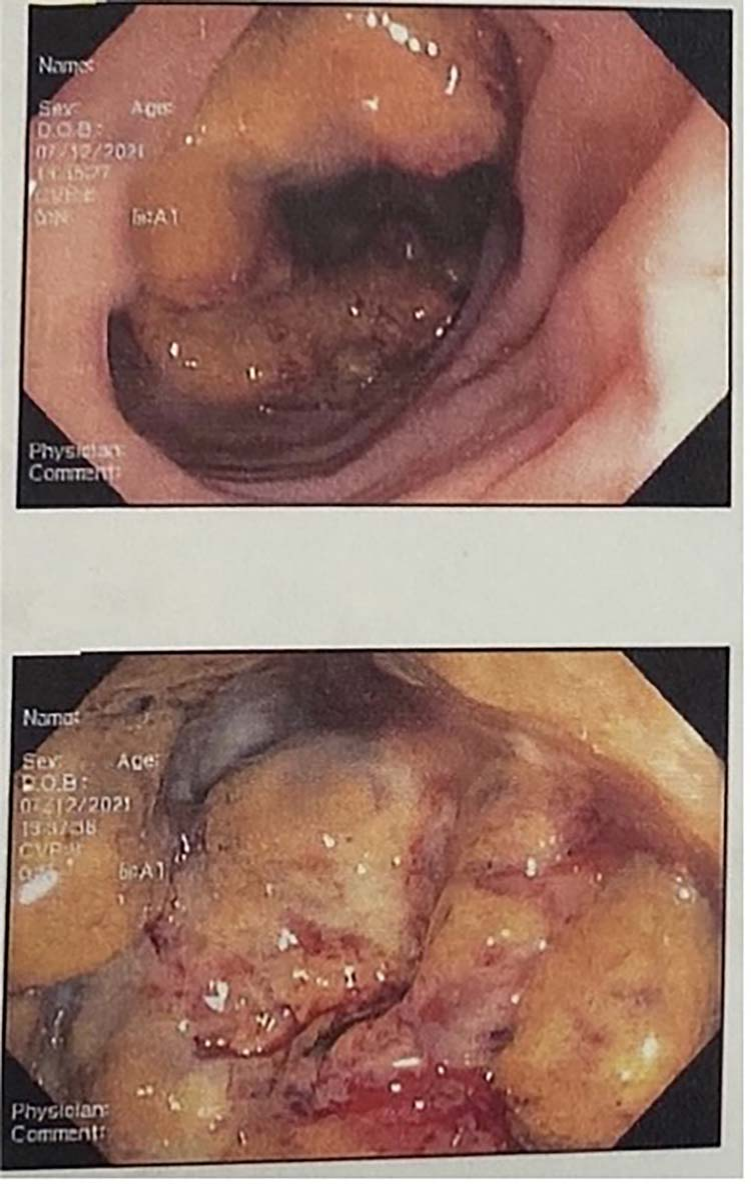
Colonoscopy shows large, necrotic, obstructive, ulcerated friable mass starting ~10 cm from the anal verge, multiple biopsies obtained at the rectosigmoid junction.

A 71-year-old female with a past medical history of Diabetes Mellitus and Hypertension, presented to the Emergency Department complaining of multiple episodes of hematochezia, generalized abdominal pain, weakness and anorexia. Significant physical examination included diffused abdominal tenderness and a digital rectal exam evidencing hematochezia. Laboratory values were significant for hemoglobin 6.3, CEA 13.7 and albumin 2.9. CT scan revealed a large mass lesion within the lower pelvis, suggestive of a neoplastic process, measuring 7.6 × 6.3 cm in diameter. Colonoscopy revealed obstructive rectal mass identified at 10 cm from the anal verge (Fig. 1). The patient was optimized before surgical intervention. Rectal mass resection, extensive lysis of adhesion and Hartmann’s procedure were performed. Hemovac drainage was left in the pelvic region.

**Figure 2:**
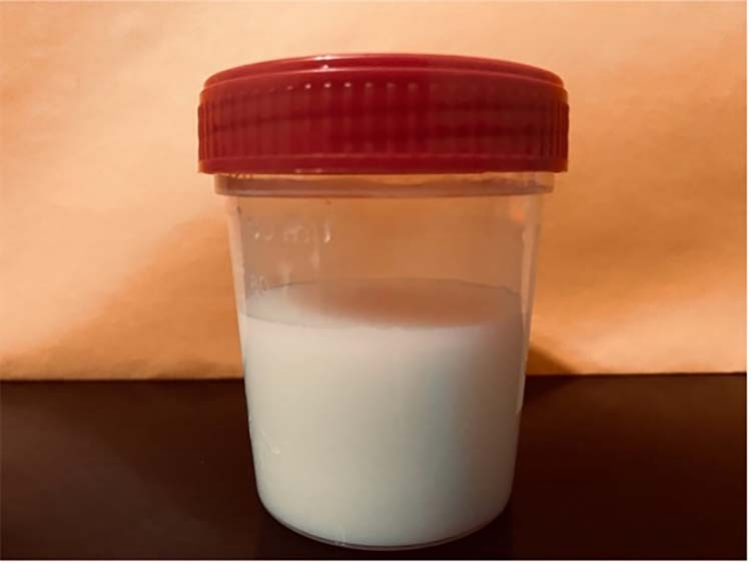
Sample of typical chylous ascites displaying cloudy and milky fluid in appearance.

Pathology confirmed a mucinous adenocarcinoma 9.3 cm in greater dimension moderately differentiated, inflammatory tumor invading through the visceral peritoneum with gross perforation. No regional lymph nodes for metastasis (0/12). Hemovac started draining milky secretions 4 days after surgical intervention (Fig. 2). CA was confirmed with a fluid sample, showing fluid triglycerides >550 mg per dL.

Diet was adjusted to a low-fat diet based on high-protein content with medium-chain triglycerides via the enteral route. Drainage output reduced significantly within 2 days post-diet adjustment.

## DISCUSSION

Some abdominal surgical procedures may cause CA [1]. CA is a rare form of ascites, known as the leakage of the lipid-rich lymph to the peritoneal cavity [2]. Damage or obstruction to the lymphatic system or one of the associated ramifications produces ascites with a turbid appearance due to the high triglyceride content [2]. Most reports on CA as a complication after surgical procedures involve patients who went through abdominal aortic surgery, pelvic surgical intervention for more complex gynecologic malignancies, or lymphadenectomy for testicular and renal cancers [3, 5]. A few reported studies have evidenced that are the extent of lymphadenectomy and tumor site and are among the risk factors for CA, especially in the right-sided colonic surgery, mostly supplied by the superior mesenteric artery, as these areas are characterized by rich lymphatics content, which may be easily damaged in the course of oncological clearance [1]. Abdominal and Pelvic CT scan can help assist diagnosis and presence of CA giving its similar density to water among other diagnoses such as Small Bowel Obstruction and intra-abdominal lesions [6].

In addition, diagnosis can be more specifically confirmed through fluid sample of CA based on the distinct characteristic of the ascitic fluid’s milky appearance and a triglyceride level of >200 mg/dL, although some authors use a cut off value of >110 mg/dL [4].

**Figure 3:**
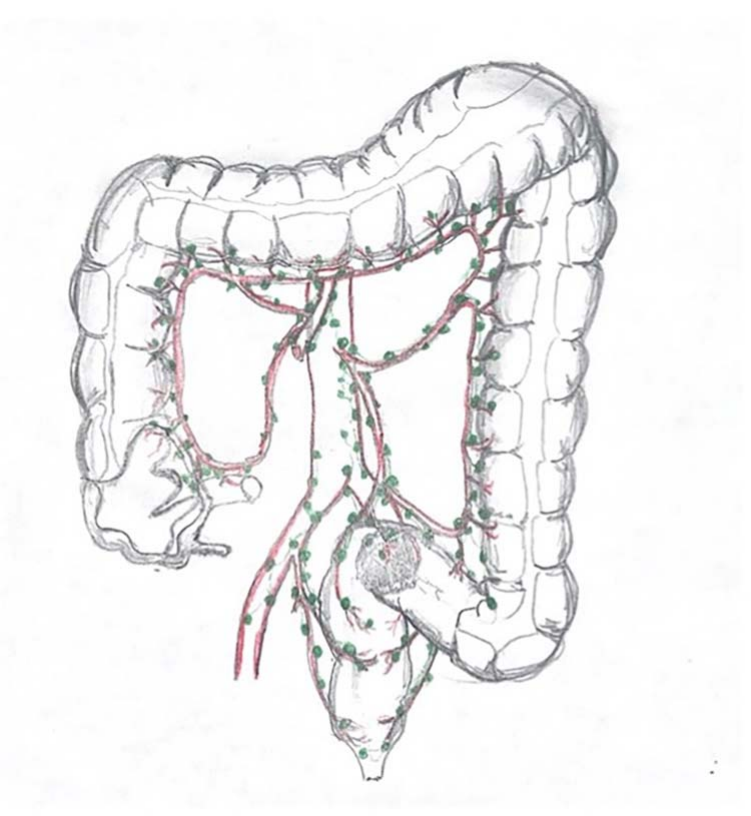
Drawing representation of the lymphatic system in large colon.

In our case during surgery, the tumor was invading the visceral peritoneum surface adjacent to the posterior uterus. Mesorectum dissection with continuous bipolar energy to fuse was difficult to perform due to the presence of inflammatory tissue; this might have resulted in damage to the lymphatic system and the milky appearance of her Hemovac output (Fig. 3). Fluid triglyceride levels were obtained in order to confirm our suspicions. The patient was treated conservatively with diet modification. The main management for this medical condition focuses on reducing ascites formation. For this purpose, a diet intake with a high-protein content, low fat, rich on median chain fatty triglycerides with long chain triglycerides restriction is used, so the conversion to monoglycerides and free fatty acids can be avoided and chyle flow reduced concomitantly. Other case reports or small observational studies describe different managements such as using drug prescription such as Orlitad in cirrhotic patients with CA as well as Somatostatin and Octreotide for those who are not responding to a conservative treatment based on diet adjustments and even those patients unresponsive to surgical intervention.

## References

[1] Soyer V, Kayaalp C. CASE REPORT postoperative chylous ascites after right hemicolectomy. European J Surg Sci 2014;5:111–3.

[2] Al-Busaf SA, Ghali P, Deschênes M, Wong P. Chylous ascites: evaluation and management. ISRN Hepatology 2014;240473:1–10. 10.1155/2014/240473 PMC4890871 27335837

[3] Qin NZ, Han M, Nien BH, Keelan S. Chylous ascites in colorectal surgery: a systematic review. World J Gastrointestinal Surgery 2021;13:585–96. 10.4240/wjgs.v13.i6.585 PMC8223702 34194616

[4] Bhardwaj R, Vaziri H, Gautam A, Ballesteros E, Karimeddini D, Wu GY. Chylous ascites: a review of pathogenesis, diagnosis and treatment, review article. J Clin Transl Hepatol 2018;6:105–13. 29577037 10.14218/JCTH.2017.00035 PMC5863006

[5] Baek S, Kim S, Kwak J, Kim J. Incidence and risk factors of chylous ascites after colorectal surgery. Am J Surg 2013;206:555–9. 23856087 10.1016/j.amjsurg.2013.01.033

[6] Moroson T, De Robles S. Chylous ascites as a marker for intestinal viability in a small bowel obstruction: a case report. J Surg Case Rep 2021;9:1–3. 10.1093/jscr/rjab411 PMC8478472 34594490

